# The Characteristics of Diverticular Disease in Caribbean Population: A Control Group Study

**DOI:** 10.1155/2022/8360837

**Published:** 2022-12-07

**Authors:** Moana Gelu-Simeon, Matthieu Schnee, Marie-Josée Lafrance, Pauline Plazy-Chabrand, Anne-Sophie Schneck, Georgette Saint-Georges, Marceline Alexis, Stéphanie Delumeau, Philippe Montigny, Roger Faroux, Jérome Dimet, Eric Saillard

**Affiliations:** ^1^Univ Antilles, Univ Rennes, INSERM, EHESP, IRSET (Institut de Recherche en Sante Environnement et Travail), UMR-S 1085, Pointe-a-Pitre F-97100, France; ^2^Service d'Hepato-Gastroenterologie, CHU de la Guadeloupe, Pointe-a-Pitre F-97100, France; ^3^Service d'Hepato-Gastroenterologie, CHD Vendee-La Roche sur Yon, la Roche sur Yon F-85000, France; ^4^Service de Chirurgie Digestive, CHU de la Guadeloupe, Pointe-a-Pitre F-97100, France; ^5^Service de Radiologie, CHU de la Guadeloupe, Pointe-a-Pitre F-97100, France; ^6^Service de Radiologie, CHD Vendee-La Roche sur Yon, la Roche sur Yon F-85000, France; ^7^Centre de Recherche Clinique, Centre Hospitalier Intercommunal de Mont de Marsan et du Pays des Sources, Mont de Marsan F-40000, France

## Abstract

**Background:**

Diverticulosis is not well characterized in the Caribbeans. Our aim was to compare the anatomical presentation of colonic diverticulosis in African Caribbeans (group AC) versus Europeans (group E) and severity.

**Methods:**

We conducted a prospective controlled study involving 274 patients admitted for lower gastrointestinal haemorrhage (LGIH) in France (center 1: Guadeloupe; center 2: La Roche-sur-Yon); 179 cases with diverticular haemorrhage, including 129 in group AC and 40 in group E. Exploration of the colon included a detailed assessment of diverticula using a dedicated endoscopic grid.

**Results:**

AC and E had similar characteristics in terms of age, gender, previous history of LGIH, body mass index, dietary habits, and medications, but AC had significantly poorer hemodynamic parameters at admission and required more blood transfusions (66.7% vs. 42.5%; *p*=0.01) during hospitalization. Out of the 169 patients included in the study, a complete exploration of the colon was achieved in 81% (*N* = 137) (AC, *n* = 106; E, *n* = 31), and revealed right-side diverticulosis in AC (in 90.6%, included into a pancolonic form in 73.6% vs. 35.5%; *p*=0.0002) and left-side diverticulosis in E (in 96.8%, isolated form in 58.1% vs. 9.4%, *p*=0.0002). These data were confirmed by a sensitivity analysis using an endoscopic grid in 92 patients, achieving a higher frequency and larger size of diverticula in AC.

**Conclusion:**

Our study has shown that diverticulosis was pancolonic in AC and more frequently associated with more severe haemorrhage than the left-sided diverticulosis of Europeans. This anatomical presentation may be driven by the genetic background more than the environment and diet.

## 1. Introduction

There are considerable geographic variations in the prevalence of colonic diverticular disease (DD). It is common in Western countries, with an estimated prevalence that ranges from 45% [1] to 60% in patients aged over 80 years [2–4]. According to prospective studies, prevalence has increased in Asian countries since the 1990s, from 12% to 25% [5, 6]. However, in Africa, there is a lack of large-scale studies to determine its exact prevalence, but DD has evolved since the first reports issued in the 1980s [7–10] and is currently emerging as an important health issue [11–13].

Several factors have been associated with an increased risk of DD and its complications, including advanced age, gender, obesity, and dietary fiber intake [6, 14–17]. Significant changes to dietary habits and improvements in life expectancy have also been proposed as explanations for the rising incidence of DD in Africa and Asia [6, 18]. In Africa, where a high-fiber diet continues to prevail, particularly in rural areas [19, 20], a gain in life expectancy is probably the main etiological factor [21].

The diverticular disease remains mainly asymptomatic; however, in 25% of individuals, it will develop as a symptomatic disease. The pathogenesis of symptomatic and complicated DD remains uncertain but involves at least in part, genetic predisposition, low-grade inflammation, altered intestinal microbiota, visceral hypersensitivity, and abnormal colonic motility [22].

Preliminary data supported that ethnicity and genetic background may be implicated in the anatomical distribution and presentation of colonic DD [17]. Diverticula are mainly located in the left colon in Europeans [23–25], while they are reported in the right colon in Asians [15, 26–30]. However, discrepancies persist regarding the localization of diverticula in Africans; some studies have reported a pancolonic distribution [11, 12], and others a right-sided [1, 14] or sigmoid localization [13]. Caribbeans are mainly of African descents but also issued from a mixed population from Europe, Africa, and Asia, due to the slave trade of population from sub-Saharan Africa and Europe between the seventeenth and nineteenth centuries. Only a few descriptive reports exist concerning the characterization of DD or its complications in the Caribbeans [14, 31–34]. Most previous studies in African descent concerned retrospective reports of uncomplicated DD, the results of screening colonoscopies [2, 10, 13, 35], or only involved a small sample of patients [11, 12]. None of these studies were performed using a controlled design and only one was prospective [1].

The present study was performed prospectively in a Caribbean population and involved a control group of Europeans. To better characterize the role of ethnicity and people migration in DD, the comparison of African Caribbeans to Europeans, because they are exposed to a similar environment (lifestyles, diet, and access to health care, at least in the French Caribbean islands thanks to the national healthcare system), could be useful.

The aim was to compare the anatomical presentation as the first endpoint and the severity of colonic DD as the secondary endpoint, both as a function of the ethnic group. Moreover, a dedicated endoscopic grid was used to obtain precise insights into DD.

## 2. Materials and Methods

### 2.1. Conduct of the Study

**Figure 1:**
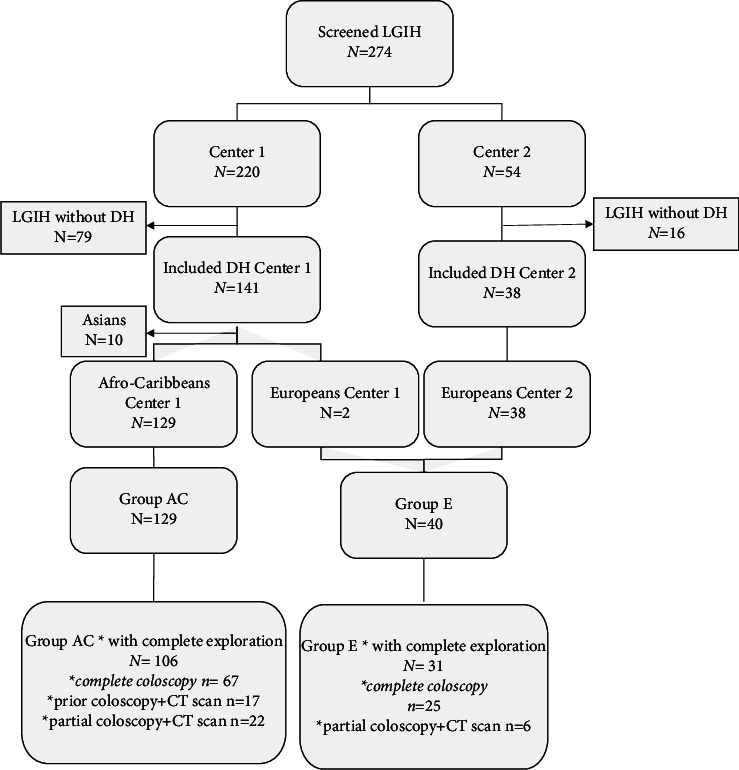
Flowchart.

This noninterventional study was conducted prospectively in the gastroenterology departments at the University Hospital of Guadeloupe (center 1) and La Roche-sur-Yon Regional Hospital, Vendée (center 2), two tertiary referral centers for gastrointestinal bleeding (Figure 1). Center 1 concerned a majority of African Caribbeans (group AC), while center 2 included exclusively Europeans (group E). The ethnic group was based on the phenotype and self-reported by the patients (African, Asian, and European).

### 2.2. Patients

Eligible patients were screened between March 9, 2013, and March 9, 2015, during which they were admitted for lower gastrointestinal hemorrhage (LGIH); this was defined as rectal bleeding and a normal upper endoscopy if the hemoglobin level was lower than 10 g/dl at admission, or melena with normal upper endoscopic findings. The included patients were 18 years or with a diagnosis of diverticular hemorrhage (DH), defined as (1) presumptive if colonic diverticula were present and no other obvious lesions could explain the bleeding or (2) definitive because active bleeding, an adherent clot, or a nonbleeding visible vessel was observed from diverticula [36]. They underwent a full exploration of the colon obtained by colonoscopy and/or a computed tomography scan (CT scan). Patients were excluded from the study if they had previously undergone a colon resection or were diagnosed as suffering from polyposis, colitis, or colon cancer during the colonoscopy. Informed consent was obtained from all participants according to French legislation regarding ethics and human research (Huriet-Serusclat law, DGS 2003/0395). The Institutional Review Board and the CNIL (DR-2013-077) approved this study, registered as no. 2012-A00949-34 by the Ethics Committee (CPP) for Bordeaux (France).

### 2.3. Endoscopic Grid

During the colonoscopy, a senior endoscopist (with more than 5 years of experience) completed a detailed endoscopic grid, which reported the size and number of diverticula in each colonic segment (right, transverse, left, or sigmoid colon and rectum). The distribution of diverticula in the colon was assigned to one of four conventional categories for the disease according to the classification developed by Golder et al. [14]: left-side DD up to the splenic flexure; right-side DD over the splenic flexure; bipolar DD as any segment involved in the right and left sides but not affecting the transverse colon, and pancolonic DD as the presence of diverticula in all colonic segments, excluding the rectum. The size of the diverticula in each segment was estimated using open biopsy forceps as being smaller, larger, or on average the same size. The number of diverticula was estimated segment by segment and stratified according to four categories: 1: no diverticula, 2: fewer than 50, 3: between 50 and 100, and 4: more than 100.

### 2.4. Colon Exploration Procedure

A CT scan was performed in patients with a hemoglobin level lower than 10 g/dl at admission. For those with a diagnosis already determined by a colonoscopy carried out within the past three years, the evaluation was considered to be complete and no additional colonoscopy was required. In the absence of these diagnostic criteria, a complete colonoscopy (satisfactory preparation and complete examination up to the cecum) was performed. For patients with an incomplete colonoscopy due to a technical failure or severe comorbidities contraindicating analgesia, exploration was completed by means of a CT scan.

The CT scans were reinterpreted by two senior radiologists specialized in abdominal radiology at each center. These two senior radiologists, SD and PM, assessed the number of diverticula in each colonic segment according to the four-point graded scale used for the endoscopic grid. The size of the diverticula on the CT scan was defined as large when greater than 1 centimeter and giant when greater than 2 centimeters [3].

### 2.5. Management Algorithm

Severe active hemorrhage was defined by a pulse rate higher than 100 bpm and/or a systolic arterial blood pressure lower than 90 mmHg and/or more than 6 units of packed red blood cells transfused during the same period of hospitalization in a context of persistent bleeding. The therapeutic strategy consisted of medical management with appropriate transfusion support and procoagulant agents associated with endoscopic hemostasis, when possible. Surgical colon resection of the diseased segment or subtotal colectomy was performed in case of more than 10 units of packed red blood cells transfused with a failure of endoscopic hemostasis.

### 2.6. Collection of Data

The following clinical and biological data were recorded at admission: age, gender, body mass index, comorbidities, past LGIH, use of anticoagulant, antiplatelet, or nonsteroidal anti-inflammatory drugs, type of bleeding (melena or rectal bleeding), systolic and diastolic blood pressure, heart rate, hemoglobin level, platelet level, and prothrombin time. During hospitalization, the numbers of packed blood cell units and hemostatic endoscopic or surgical procedures were recorded. The average daily consumption of fruits, vegetables, and whole grain food was assessed following a qualitative dietary questionnaire prospectively completed by a dietitian during a face-to-face patient interview. This questionnaire assessed the frequency of fruits, vegetables, and whole grain foods (bread, pasta, and rice) usually consumed per day, per week, or per month and was completed in the two centers. The starchy foods, a representative of carbohydrate intake, were also reported qualitatively and compared in the two populations, as starchy foods are quite popular in the Caribbean islands' eating habits.

### 2.7. Statistical Analysis

The statistical analysis was performed using SAS software (version 9.4, SAS Institute Inc., Cary, NC, USA). Means and standard deviations were calculated for continuous variables, medians for skewed distributions of continuous variables, and proportions for categorical data. We determined the distribution of diverticula at the group level. Spearman's correlation was used to assess the association between participant characteristics and the distribution of diverticula by location. We estimated odds ratios and 95% confidence intervals using the nonparametric Mann–Whitney test for quantitative variables, while the chi-square test or Fisher's exact test was used for qualitative variables. All tests of significance were two-tailed, and *p* values <0.05 were considered to be significant. All authors had access to the study data and reviewed and approved the final manuscript.

## 3. Results

### 3.1. Study Population

Overall, 274 patients were screened for LGIH: 220 in center 1 and 54 in center 2 (Figure 1). Of them, 179 patients with DH were included: 141 in center 1 and 38 in center 2, including 129 (72.1%) in group AC and 40 (22.3%) in group E. Ten Asian patients were excluded from the analysis.

Similar characteristics were found in groups AC and E with respect to age, gender, previous history of LGIH, body mass index, dietary habits, and the prescription of platelet aggregation inhibitors, oral or subcutaneous anticoagulants, antihypertensive, antidiabetic, or non-steroidal anti-inflammatory drugs (Table 1). Vitamin K antagonists were significantly more prescribed in Europeans (35% vs. 11.6%, *p*=0.001). Median PT ratios and platelet counts did not differ between the two groups.

African Caribbeans had significantly lower systolic blood pressure values (117 (100–130) vs. 127 (110–138); *p*=0.02), a higher proportion of those with a high pulse rate (higher than 100 bpm in 28% vs. 5%; *p*=0.001), and a higher proportion of nadir hemoglobin rates lower than 8 g/dl (42.6% vs. 22.5%; *p*=0.03) or those requiring blood transfusions (66.7% vs. 42.5%; *p*=0.01) than Europeans.

### 3.2. Management of Diverticular Hemorrhage

Almost all patients were managed medically, i.e., 123 (95.4%) vs. 40 (100%), *p*=0.19, in groups AC vs. E, respectively. Moreover, DH was treated endoscopically in three patients (2.33%) vs. 0 (0%), *p*=0.44, or surgically in eight patients (6.2%) vs. 1 (2.5%), *p*=0.32, respectively, and no radiological embolization procedures were performed. Surgical management consisted in one right hemicolectomy (CT scan with contrast blush), two left hemicolectomies, and five subtotal colectomies (severe hemorrhage without contrast blush) in group AC, vs. one left hemicolectomy in group E.

### 3.3. Diverticular Extension

As shown in Figure 1, a complete exploration of the colon was assessed in 106 (82.2%) patients from group AC and 31 (77.5%) from group E. This was achieved using (1) a complete colonoscopy in 84 patients from group AC (79.2%; *n* = 67 during hospitalization, *n* = 17 during the three years before hospitalization) and 25 patients from group E (80.6%); or (2) a partial colonoscopy associated with a CT scan or virtual colonoscopy in 22 (20.8%) and 6 (19.4%) patients, respectively.

Diverticula were significantly more observed, in the right-side colon in group AC than in group E (Table 2). It concerned, respectively, 90.6% vs. 41.9% of patients; *p* < 0.0001; including 73.6% vs. 35.5% pancolonic forms; *p*=0.0002, 8.5% vs. 3.2% isolated right-side DD; *p*=0.03, and 8.5% vs. 3.2% bipolar forms; *p*=0.03. Inversely, in group E, diverticula were localized in the left-side colon in 96.8% of patients, including isolated left-side DD (in 58.1% vs. 9.4%, *p*=0.0002) in the majority of cases (Table 2).

In the sensitivity analysis of 92 patients undergoing a complete exploration of the colon using the dedicated endoscopic grid (Table 3), we still observed higher frequencies of right-side DD (89.6% vs. 40%, *p* < 0.0001), mainly included into pancolonic DD (in 71.6% vs. 32%, *p* < 0.001) in group AC and isolated left-side DD in group E (in 60 vs. 10.4%, *p*=0.002). There was no difference in the presence and number of diverticula in the right-side and in the left one in AC, while Europeans were clearly characterized by left colon diverticula (Table 3).

The number of patients with more than ten diverticula in the left colon (61.2% vs. 32%, *p*=0.02) or more than 100 diverticula in one segment (70.1% vs. 3.8%, *p* < 0.0001) was significantly higher in group AC vs. group E (Table 3). Moreover, in the right colon, the highest frequency of diverticula was obtained in the class (50–100), with a median class calculated at 65 diverticula for group AC vs. 14 diverticula (median class (0–50)) in group E. In the left colon, the highest frequency of diverticula was obtained in class (50–100), with a median class calculated at 84 diverticula for group AC vs. 26 diverticula (median class (0–50)) in group E. The diverticula were also significantly larger in the group AC (more than 2 cm in 23.9% vs. 0%; *p*=0.004).

**Figure 2:**
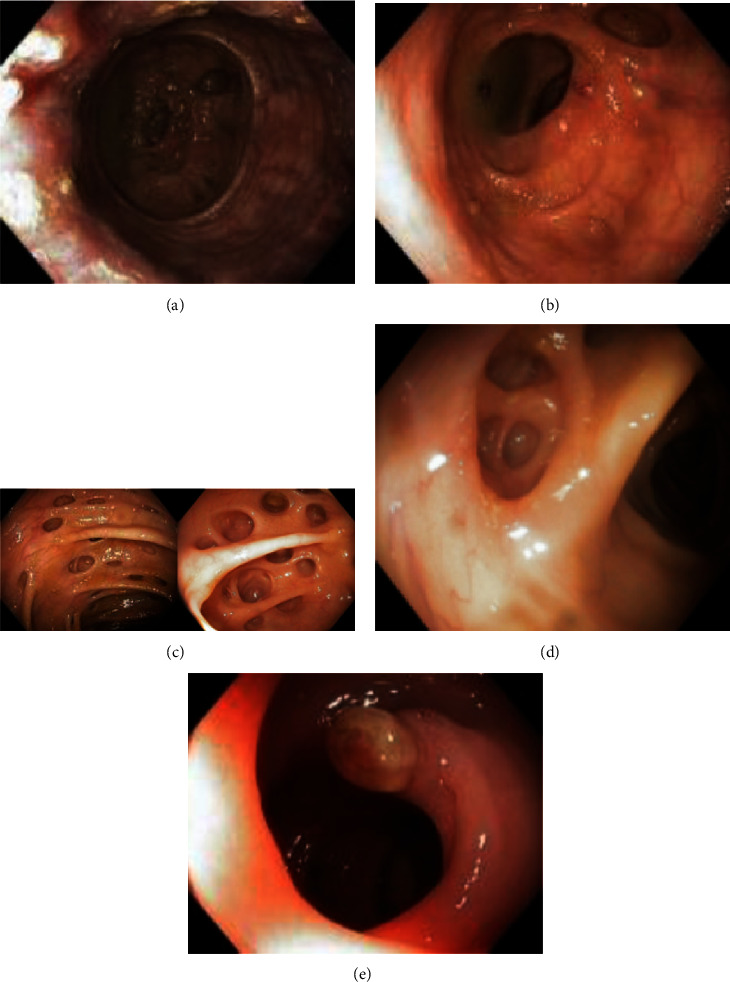
Pancolonic diverticulosis in a 50-year-old African Caribbean patient consisting of both a large rectal diverticulum (a), rare diverticula in the left-side (b), multiple (c) and deep second and third generation diverticula (d) in the right-side colon and an inverted diverticulum (e).

Interestingly, the endoscopist noted a nonplanned criterion that was recorded in African Caribbean patients: second (30/67 patients, 44.8%) and third (11/67 patients, 16.4%) generations of diverticula developed deeper in the colonic wall were observed (illustrated in Figure 2), whereas this was not analyzed in Europeans.

## 4. Discussion

This is the first prospective study to have used a dedicated endoscopic grid to compare DD in African Caribbeans versus a control group of Europeans. We observed that African Caribbeans had a mainly pancolonic form of DD with no significant difference in the presence and number in the right-side and left-side diverticula. Conversely, in Europeans, diverticula were mainly located on the left colonic side and less abundant and smaller than in AC.

There was no difference according to age, gender, previous history of LGIH, body mass index, dietary habits, or medications, but the clinical and endoscopic presentation of DD was significantly more severe among African Caribbeans than Europeans.

Only one American prospective study [1] had previously reported endoscopic data in African Americans undergoing a screening colonoscopy. They included 260 patients with diverticula, 20% of African Americans, and reported a significantly higher proportion of patients with diverticula in the right-side colon among African Americans (20% of them) compared with other ethnical groups. Golder et al. [14] also reported in their retrospective study on double contrast barium enema that African descents (including African Caribbeans) were up to three times more likely to have a right-side localization of diverticula compared with Europeans. Moreover, pancolonic forms of DD have been mainly reported by retrospective African studies, with rates ranging from 10% [13] to 85% of patients [10–12, 35]. This characteristic was probably conserved following the migration of African populations and is mainly due to genetics as opposed to the environment.

The risk of having a higher number of diverticula among African descent than Europeans was controversial between the two studies, according to the studies of Golder et al. [14] and Peery et al. [1]. In our study, a significantly higher proportion of patients had more than 100 diverticula per segment (70.1% vs. 3.8%, *p* < 0.001) and the median classes of diverticula were also higher in African Caribbeans than Europeans, in both the right and left-sides of the colon. These data confirm a tendency toward a higher proportion of diverticula in the colon of African Caribbeans, whatever the segment.

Numerous questions remain regarding the genesis of DD. Some studies have shown that changes to the colonic wall may affect the pathogenesis of colonic DD [37]. Changes to colonic tenseness, as manifested by lower levels of collagen and elastin in the colonic wall associated with the ageing process, support this view [17, 38]. Because our study population was slightly older than those in previous studies, this could explain the overrepresentation of pancolonic DD in our cohort. Moreover, diverticulosis and diverticulitis are very common in patients with the Ehlers-Danlos syndrome and Marfan syndrome [39], which suggests that changes to collagen in the intestinal wall might also predispose patients to DD. Large genome-wide association analyses on DD identified diverticulosis risk loci that contain genes involved in connective tissue integrity and intestinal motility, highlighting the importance of neuromuscular abnormalities in the development of diverticulosis. However, these data have been obtained only on individuals of European descent [40, 41], and currently, there are no data on African descent.

Nevertheless, it might be interesting to conduct a study on the structural characteristics of supportive connective tissue and their genetic background in populations from different ethnic groups.

The clinical severity of DD has been evaluated in three retrospective studies that included 30 to 40 Africans, and DH was the most common complication (in more than 50%) [11–13]. Estimates of the prevalence of DH requiring hospitalization were also obtained from a nationwide inpatient sample [3], and African Americans experienced a higher prevalence than their European counterparts. There also might have been structural effects that disproportionately affected African Caribbean patients, even if everybody theoretically had the same access to healthcare. Further, the source of bleeding was more likely to be diverticula in the right-side colon than in the left-side. Indeed, it has been suggested that right-side diverticula that involve all three layers of the colonic wall might be more likely to bleed; arterial bleeding tends to be more profuse, and the wall of the right colon is thinner than that on the left, while the erosion of a vessel from the collar of a diverticulum also seems more likely to bleed, particularly when the diverticulum is large [28, 29]. Surprisingly, in Asians, among whom there is also a tendency for right-side diverticula, the prevalence of DH remains globally low (1.5% of all colonic diverticula reported by Nagata et al. [5]) and occurs preferentially in cases of bilateral DD (47% of bleeding cases). More recently, Imaeda and Hibi [15] also reported that both left- and right-sided diverticulosis in Asians, and right-sided in Western countries, increased the risk of bleeding. Faucheron et al. [24] reported that pancolonic diverticulosis was associated with a significant risk of bleeding, independently of patient age. Controversies therefore persist concerning the origin of bleeding, but it was not surprising in our study that as a result of colon involvement, DH cases were more severe in African Caribbeans than Europeans.

## 5. Conclusions

Our study has shown a right-sided involvement and pancolonic forms of diverticula in African Caribbeans compared to the left-sided localization in Europeans, and particularly the greater endoscopic and clinical severity of DH in African Caribbeans. This particular presentation is probably related to the genetic background of their African ascendance as opposed to the environment. Patient management should be adapted to this particular presentation of DD in African Caribbeans, and the possibility of more frequent subtotal colectomies should be discussed in cases of severe hemorrhage. In view of the fact that DH occurs most frequently in elderly patients with comorbidities and multiple medications, it is now essential to determine specific prognostic markers for this condition.

## Figures and Tables

**Table 1:** Characteristics of patients included in the study.

Characteristics	Group AC *N* = 129	Group E *N* = 40	*p* value
Male (*n*, %) (95% CI)	81 (62.8%) (53.8–71.1)	27 (67.5%) (50.9–81.4)	0.7
Age (median, IQR)	78 (70–104)	80 (74–96)	0.3
BMI (kg/m^2^) (median, IQR)	25.4 (23.4–28)	26 (24.2–29.7)	0.2
*Dietary fiber intake*			
Fruit < 1/day (*n*, %) (95% CI)	118 (91.5%) (85.2–95.7)	38 (95%) (83.1–99.4)	0.7
Vegetable < 1/day (*n*, %) (95% CI)	113 (87.6%) (81.8–93.2)	38 (95%) (83.1–99.4)	0.24
Whole grain food < 1/day (n, %) (95% CI)	99 (76.7) (69.4–84.0)	33 (82.5) (70.7–94.2)	0.442
Starchy food intake > 3/day (n, %) (95% CI)	25 (19.4%) (12.6–26.2)	6 (15%) (5.7–29.8)	0.7
Previous LGIH (*n*, %) (95% CI)	41 (31.8%) (23.7–39.7)	11 (27.5%) (14.6–43.9)	0.75
*Anticoagulants*			
Vitamin K antagonist (*n*, %) (95% CI)	15 (11.6%) (6.7–18.4)	14 (35%) (20.6–51.7)	**0.001**
Heparin (*n*, %) (95% CI)	2 (1.6%) (0.2–5.5)	0	1
DOAC (*n*, %) (95% CI)	5 (3.9%) (1.3–8.8)	0	0.59
Antiplatelet drugs (n, %) (95% CI)	60 (46.5%) (37.7–55.5)	22 (55%) (39.6–70.4)	0.44
NSAIDs (*n*, %) (95% CI)	13 (10.8%) (5.5–16.6)	3 (7.5%) (0–15)	0.76
PT ratio (median, IQR)	81 (66–91.5)	76 (47–90.5)	0.5
Platelet count (G/L) (median, IQR)	219 (172–271)	205 (174–260)	0.46
SBP (mmHg)^*∗*^	117 (100–130)	127 (110–138)	**0.02**
SBP ≤ 90 (mmHg)^*∗*^ (*n*, %) (95% CI)	21 (16.3%) (10.4–23.8)	3 (7.5%) (0–15)	0.2
PR^*∗*^ (median, IQR)	90 (77–102)	87 (68–84)	**0.0001**
PR > 100/min^*∗*^ (*n*, %) (95% CI)	36 (28%) (20.2–35.8)	2 (5%) (0.6–17)	**0.001**
Nadir Hb level < 8 g/dl (*n*, %) (95% CI)	55 (42.6%) (34.1–51.1)	9 (22.5%) (10.8–38.4)	**0.03**
Patients transfused (n, %) (95% CI)	86 (66.7%) (57.8–74.7)	17 (42.5%) (27–59.1)	**0.01**

Group AC, African Caribbeans; group E, Europeans; BMI, body mass index; LGIH, lower gastrointestinal hemorrhage; NSAIDs, nonsteroidal anti-inflammatory drugs; SBP, systolic blood pressure; PR, pulse rate; Hb, hemoglobin; DOAC, direct acting oral anticoagulant. ^*∗*^data at admission. Quantitative values are expressed as median, IQR. Qualitative values are expressed as *n*, % (95% CI).

**Table 2:** Complete exploration of the colon in African Caribbean and European patients.

Complete exploration	Group AC with complete exploration *N* = 106	Group E with complete exploration *N* = 31	OR (95% CI)	*p* value
Pancolonic DD	78 (73.6)	11(35.5)	5.06 (2.15–11.88)	**0.0002**
Right-side DD^*∗*^	9 (8.5)	1 (3.2)	9 (1.03–78.17)	**0.03**
Left-side DD^*∗*^	10 (9.4)	18 (58.1)	0.06 (0.01–0.32)	**0.0002**
Bipolar left and right-side DD^*∗∗*^	9 (8.5)	1 (3.2)	9 (1.03–78.17)	**0.03**

Group AC, African Caribbeans; group E, Europeans; DD, diverticular disease. Qualitative values are expressed as *n*, %. ^*∗*^without diverticula in controlateral side. ^*∗∗*^without diverticula in transverse colon.

**Table 3:** Complete exploration of the colon in African Caribbean and European patients using a dedicated endoscopic grid.

Complete exploration with endoscopic grid	Group AC with endoscopic grid *N* = 67	Group E with endoscopic grid *N* = 25	OR (95% CI)	*p* value
*Extension*				
Pancolonic DD	48 (71.6)	8 (32)	5.36 (1.98–14.5)	**0.001**
Right-side DD^*∗*^	9 (13.4)	1 (4)	14 (1.57–131.52)	**0.008**
Left-side DD^*∗*^	7 (10.4)	15 (60)	0.07 (0.01–0.44)	**0.002**
Bipolar left and right-side DD^*∗∗*^	3 (4.5)	1 (4)	3 (0.28–32)	0.6
Severity				
*Number*				
>10 in left-side	41 (61.2)	8 (32)	3.35(1.26–8.87)	**0.02**
>100 in one segment	47 (70.1)	1 (3.8)	58.75 (7.44–463.81)	**<0.001**
Size >2 cm	16 (23.9)	0 (0)	NA	**0.004**

Group AC, African Caribbeans; group E, Europeans; NA, not applicable. Qualitative values are expressed as *n* (%). ^*∗*^without diverticula in controlateral side. ^*∗∗*^without diverticula in transverse colon.

## Data Availability

The data used to support the findings of this study are available from the authors upon request.

## Abbreviations

CT scan: Computed tomography scan
DD: Diverticular disease
DH: Diverticular hemorrhage
Group AC: African Caribbean
Group E: European
LGIH: Lower gastrointestinal hemorrhage.
